# A Climax at Torbay Hospital

**Published:** 1902-04-26

**Authors:** 


### A CLIMAX AT TORBAY HOSPITAL.

The dispute between the medical men and the weekly board of Torbay Hospital has now come to a climax. It will be remembered that the dispute arose concerning the method of election of the honorary medical staff, this being precipitated by the removal of two doctors from the list. After a particularly heated meeting, an adjournment was made for a conference between the board of management and the Torquay Medical Council, with a view to settling the difficulty; but this unhappily proved abortive, for while the committee were ready to consent to withdraw the objectionable rule which provided for annual election, the medical men were unwilling to accept the olive branch unless the concession was made retrospective—in other words, that their two *confrères* should be reinstated—and carried a resolution accordingly. On Monday, April 21st, the Governors of the hospital met to consider the decision of the conference and resume consideration of the proposal that the rule providing for the annual election of medical staff be repealed, and that the medical staff hold office until some cause arises requiring their resignation. Mr. J. Kitson presided. It soon became evident that the managing board regarded the action of the medical men as a vote of censure on them, for Mr. Bowring announced that the chairman and the secretary had resigned, and he intended to follow their example. He declared that it was impossible in view of this conflict between the medical men and the board that things could go on harmoniously, for the latter had been told that they had perpetrated irregularities, injustices, illegalities, and if he remembered rightly, one member of the medical profession had stated that their conduct had been iniquitous. Such epithets hurled against them broadcast were more than honourable men could submit to. The only practical solution that he saw was that the board should resign and that the Governors should appoint a fresh board. Mr. R. Andus Clark (the chairman of the weekly board) reviewed the controversy and declared that no self-respecting board could assent to accept the proposal to make the rescission of the rule retrospective. He refused to be coerced by what he termed a boycott, and left the platform, followed by several other members of the board, including Mr. Grant Morris, Captain Withington, Dr. Orfeur, and Mr. H. Evans. Colonel Spragge then took up the cudgels for the weekly board, declaring that some of the young blood of the profession at the conference were there no doubt anxious to get notoriety at any price. He went on to say that there was never the slightest prospect of a compromise, and said the board would let the obnoxious rule go so long as the medical men would give them some guarantee that the hospital would be guarded against deterioration or neglect. That, they were told, had stirred the medical profession to its greatest depths. He declared, amid cries of "Time" and "Business," that if the board died it would be more at the hands of the ladies than anyone else, and then announced his own resignation, remarking that the best that they could do would be to hand the hospital over entirely to the doctors. Dr. Odell defended Drs. Finch and Pollard, declared that there was no justification for their removal, and said that Dr. Jones had written to 43 hospitals and 40 had no such rule as the Torbay Hospital. The Chairman declined to accept Dr. Odell's amendment that the altered rule, if carried, should be retrospective. Dr. Stabb asked who were the people who were boycotting the hospital but the thin-skinned gentlemen who had left the platform, and was met by Mr. Curzon, who retorted: "Is not the medical profession the greatest boycotting profession in the United Kingdom?" Colonel Stovell and Mr. J. B. Guyer at this stage left the platform and left there only the Chairman, Mr. F. P. T. Struben, and Mr. R. L. Butland, and, after further discussion, the Chairman appealed to the Governors to maintain their own action and rules, and put the motion for the rescission of the rule objected to by the medical men and the substitution for it of the following:—"The honorary staff shall consist of three physicians, three surgeons, an ophthalmic surgeon, an anæsthetist, a surgeon in charge of the X-ray department, and a dental surgeon. They shall be elected as vacancies occur by a special Board, fifteen days' notice of the intention to hold an election being published in a Torquay newspaper." Some ill-feeling was caused by his refusal to accept a motion for adjournment, and then the motion was carried by 47 votes to 26, and the meeting broke up in confusion.

